# Progress in Neurology

**Published:** 1901-01-19

**Authors:** 


### PROGRESS IN NEUROLOGY.

(*Continued from page* 264.)

**Insanity in Lead Workers.**—From a large lunacy experience Jones[11] comes to the following conclusions as to the existence of lunacy in lead workers. Lead poisoning is a contributory factor in the causation of insanity, and there is a higher average number of general paralytics than in others of the population. There is a tendency in these cases to cardiac, renal, and arterial degeneration, with complications due to syncopal or epileptiform fits. Most cases present marked signs of anæmia and ill-health, with unsteadiness of gait and general impairment of muscular strength, and very frequently a history of temporary failing vision. The mental symptoms may be grouped among one or other of the following varieties:—(*a*) Those in the nature of a toxæmia with sensory disturbances, and which tend rapidly to get well. (*b*) Those with hallucinations of sight and hearing, more chronic in their nature and which may be irrecoverable. The delusions in this class are almost invariably those of being poisoned or followed about, and are in the main persecutory. (*c*) Those resembling general paralysis with tremors, increased knee-jerks, inco-ordination, and accompanied with listlessness amounting to profound dementia, but which tend to get well. In most lead cases presenting mental symptoms the tendency is to recovery unless the patient dies early.

**The Results of the Treatment of Epileptics in Colonies** has been discussed by Turner,[12] who is in charge of the epileptic colony at Chalfont St. Peter, Bucks. The epileptic is there removed from town or city environment, and is set to regular employment in garden, fields, orchards, or workshops under the supervision of capable persons. His life is well ordered and regulated, with an absence of excitement and alcohol. He has abundance of simple nourishment. Medicinal remedies are reduced to a minimum, viz. 30 grains of potassium bromide at bedtime. The effects of this are:—(*a*) The frequency of fits is usually considerably diminished, though in a minority there is at first an increase, probably due to diminution of the potassium bromide with which they were saturated on arrival. In the majority of cases an amelioration in the severity of attacks has been seen. In a very small minority there has been a steady downward tendency towards dementia associated with increased severity and frequency of the fits. But in the great majority of cases this is not seen, and many of the colonists are capable of work requiring individual alertness and tact, whilst most are able to do good work under supervision.

**Chloretone** is said by Wade[13] to be a safe and efficient hypnotic. It is but slightly soluble in water, but is in alcohol, and may be given in sherry wine or whisky. The initial dose should be 15 grains, and be gradually increased until the desired effect is produced. The pulse rate is slightly decreased, but the action of the heart is not disturbed; the blood pressure remains unaffected and the respiration shows no change. It is most valuable in the motor excitement of acute maniacal attacks and of agitated melancholia. It could very probably be used with benefit in the motor excitement of general paresis. As a hypnotic it is not so dangerous as sulphonal, which is a depressant, and it is more agreeable to take than paraldehyde, and does not leave bad after-effects.

**Dormiol** is praised by Këtly[14] as a sure and harmless hypnotic. Chemically it is dimethylethylcarbinol chloral. It is a colourless oily liquid, which forms with water a milky emulsion—the most convenient mode of administration—or it can be given in gelatine capsules. In the majority of patients it acts well in a dose of 7½ minims, and in only one or two cases had the dose to be increased to 15 or 30 minims. Sleep was induced generally after thirty or forty minutes, and lasted from four to six hours. There was no harmful effect on the heart, respiration, or temperature. It is a pure hypnotic, and does not act in the sleeplessness due to pain.

**Hypnotic Suggestion.**—Tuckey records two cases of agaraphobia cured by hypnotic suggestion. The first case was that of a lady, aged 26, who for two years had noticed that she felt nervous in church and uncomfortable in shops. Bromides, tonics, and rest from her work as a school teacher proving unsuccessful, and the symptoms growing worse, she was hypnotised, and suggestions made that she would gain confidence and lose her morbid fears. In two weeks the symptoms she had experienced in a crowded building, such as faintness, giddiness, palpitation of the heart, and a stifling sensation in the chest, with a vague terror she could not control, entirely disappeared, and she was able to resume her employment in thorough good health. The second case was that of a man, aged 37 years, strong, healthy-looking, and with a family. He had influenza badly in October 1899, but made a good recovery. He was perfectly well except for his "nerves": he was unable to go about alone, and had to be accompanied to and from his office. He could not enter a theatre or church without feeling such depression and terror that he was forced to retire. These symptoms affected his spirits and kept him awake at night, so that his health was failing. Under the influence of hypnotism he passed into the cataleptic stage and was very susceptible to suggestion. He was hypnotised daily for a week and twice afterwards. Under this treatment he was completely cured. The value of hypnotic treatment is very great in this class of case if the patient is at all hypnotisable.

[11] Brit. Med. Jour., Sept. 22, 1900. [12] Ibid., Sept. 29, 1900. [13] Jour. of Nerv. and Ment. Dis., Aug. 1900. [14] Thër. der Gegenwart, Aug. 1900. [15] Edin. Med. Jour., July 1900.

